# Early Experience after Developing a Pathology Laboratory in Malawi, with Emphasis on Cancer Diagnoses

**DOI:** 10.1371/journal.pone.0070361

**Published:** 2013-08-07

**Authors:** Satish Gopal, Robert Krysiak, N. George Liomba, Marie-Josephe Horner, Carol G. Shores, Noor Alide, Steve Kamiza, Coxcilly Kampani, Fred Chimzimu, Yuri Fedoriw, Dirk P. Dittmer, Mina C. Hosseinipour, Irving F. Hoffman

**Affiliations:** 1 Institute of Global Health and Infectious Diseases, University of North Carolina, Chapel Hill, North Carolina, United States of America; 2 Program in Global Oncology, Lineberger Comprehensive Cancer Center, University of North Carolina, Chapel Hill, North Carolina, United States of America; 3 UNC Project-Malawi, Lilongwe, Malawi; 4 Department of Epidemiology, University of North Carolina, Chapel Hill, North Carolina, United States of America; 5 Malawi Surgical Initiative, Lilongwe, Malawi; 6 Department of Otolaryngology, University of North Carolina, Chapel Hill, North Carolina, United States of America; 7 Kamuzu Central Hospital, Lilongwe, Malawi; 8 University of Malawi College of Medicine, Blantyre, Malawi; 9 Department of Pathology and Laboratory Medicine, University of North Carolina, Chapel Hill, North Carolina, United States of America; 10 Department of Microbiology and Immunology, University of North Carolina, Chapel Hill, North Carolina, United States of America; University of Massachusetts Medical School, United States of America

## Abstract

**Background:**

Despite increasing cancer burden in Malawi, pathology services are limited. We describe operations during the first 20 months of a new pathology laboratory in Lilongwe, with emphasis on cancer diagnoses.

**Methods and Findings:**

We performed a cross-sectional study of specimens from the Kamuzu Central Hospital pathology laboratory between July 1, 2011 and February 28, 2013. Patient and specimen characteristics, and final diagnoses are summarized. Diagnoses were categorized as malignant, premalignant, infectious, other pathology, normal or benign, or nondiagnostic. Patient characteristics associated with premalignancy and malignancy were assessed using logistic regression. Of 2772 specimens, 2758 (99%) with a recorded final diagnosis were included, drawn from 2639 unique patients. Mean age was 38 years and 63% were female. Of those with documented HIV status, 51% had unknown status, and 36% with known status were infected. Histologic specimens comprised 91% of cases, and cytologic specimens 9%. Malignant diagnoses were most common overall (n = 861, 31%). Among cancers, cervical cancer was most common (n = 117, 14%), followed by lymphoma (n = 91, 11%), esophageal cancer (n = 86, 10%), sarcoma excluding Kaposi sarcoma (n = 75, 9%), and breast cancer (n = 61, 7%). HIV status was known for 95 (11%) of malignancies, with HIV prevalence ranging from 9% for breast cancer to 81% for cervical cancer. Increasing age was consistently associated with malignancy [bivariable odds ratio 1.24 per decade increase (95% CI 1.19–1.29) among 2685 patients with known age; multivariable odds ratio 1.33 per decade increase (95% CI 1.14–1.56) among 317 patients with known age, gender, and HIV status], while HIV infection and gender were not.

**Conclusions:**

Despite selection and referral bias inherent in these data, a new pathology laboratory in Lilongwe has created a robust platform for cancer care and research. Strategies to effectively capture clinical information for pathologically confirmed cancers can allow these data to complement population-based registration.

## Introduction

Sub-Saharan Africa is experiencing an escalating cancer burden, as a result of the HIV epidemic, growth and aging of the population, and adoption of ‘westernized’ lifestyles [1]–[4]. For patients with cancer throughout the region, scarcity of pathology services has often been an obstacle toward receiving appropriate diagnosis and treatment. Pathologist availability in the region is typically less than one per million population versus more than 60 per million population in the United States [5]–[7].

In Malawi, a country of approximately 16 million people in southern Africa, availability of resources for cancer diagnosis is similar to the region as a whole, with pathology services until recently having been restricted to Blantyre, the country's second largest city. Lilongwe, the capital and major population center with a population of approximately one million residents, was conversely without a functioning pathology laboratory, leading to extreme diagnostic delays and complete diagnostic failures for most cancer patients. These factors contributed directly to late diagnoses for Malawians with cancer, who presented typically with advanced tumors and experienced consequently poor outcomes.

In July 2011, a longstanding collaboration between the Malawi Ministry of Health, Kamuzu Central Hospital (KCH), and the University of North Carolina at Chapel Hill (UNC), resulted in the opening of the first diagnostic pathology laboratory in Lilongwe. In this paper, we report our experience during the first 20 months of operation. Increasing pathology services is an essential component of future cancer control programs in sub-Saharan Africa,[7] and we describe our unique experience initiating pathology services *de novo* in a major city in the region. Despite inherent selection and referral bias in these data, which are discussed at length, we believe our early experience can inform strategies for improving pathology availability in settings in sub-Saharan Africa where it is currently unavailable, as well as provide insights for cancer control efforts more generally.

## Materials and Methods

### Ethics statement

This research was approved by the University of North Carolina institutional review board and the Malawi National Health Sciences Review Committee. Due to its designation as secondary analysis of existing or non-research data, a waiver of informed consent was granted by both reviewing bodies in accordance with United States 45 Code of Federal Regulations 46.116(d).

### Laboratory development and diagnostic procedures

A timeline of events in the development of the laboratory is shown in Table 1. Unused space in an existing dermatology unit was donated by KCH. UNC resources were provided for renovations, including minor structural modifications, as well as installation of electricity, plumbing, and equipment totaling more than 200,000 US dollars. New equipment for the laboratory included stainless steel benches, sinks, Perspex safety cabinet, Leica tissue processor and embedding station, microtome, ultralow freezer, and Leica microscope equipped with digital camera and computer. An Aperio^TM^ virtual microscopy system was installed, so that UNC pathologists can provide long-distance consultation as needed. A weekly telepathology session is now held with participation of clinicians and pathologists both at UNC and in Malawi, to review specimens of interest. Support for these activities was provided by the US National Institutes of Health (NIH) through the Medical Education Partnership Initiative (MEPI), the AIDS Malignancy Consortium (AMC), and the Division of AIDS (DAIDS). Additional support was provided by the UNC Lineberger Compehensive Cancer Center, and the UNC Department of Pathology and Laboratory Medicine. KCH provides the majority of consumable supplies for ongoing operations.

**Table 1:** Timeline for development of pathology services at Kamuzu Central Hospital (KCH).

Years	Progress
**Before 1981**	Specimens sent to St. Thomas' Hospital in the United Kingdom
**1982–1995**	Specimens sent to Queen Elizabeth Central Hospital in Blantyre after the arrival of Dr. George Liomba
**1995–2011**	Specimens sent to the University of Malawi College of Medicine in Blantyre
**2010–2011**	Acquisition of laboratory space at KCH in Lilongwe, renovations, equipment installation, and validation of procedures
**2011–2013**	KCH laboratory operational in Lilongwe
**2013**	Manual immunohistochemistry and digital telepathology implemented

The laboratory became operational in July 2011. Specimen review and laboratory direction are provided by Professor George Liomba, a senior Malawian pathologist trained in the United Kingdom. During the first 20 months of operations, Professor Liomba reviewed more than 70% of all specimens, and more than 95% of all specimens after joining the laboratory full-time in October 2012. Before then, specimens were sent to Professor Liomba in Blantyre where he was previously based before moving to Lilongwe. Volunteers from Pathologists Overseas additionally provided diagnostic interpretation during the early period. Malawian histology and cytology technicians were trained in Blantyre and also at the University of Witwatersrand in South Africa, and have returned home to staff the laboratory. Immunohistochemistry (IHC) is slowly being implemented (Table 2), to provide data which can be used for clinical decision making, as well as for research purposes and to support enrollment into clinical trials. UNC pathologists are able to provide real-time feedback on quality of the staining procedures as these are implemented in Malawi. During the period reported in this paper, pathologic diagnoses were based on morphology alone without the assistance of IHC, flow cytometry, or molecular diagnostic tools. Operating procedures, as well as quality assessment and control systems have been established, and include weekly telepathology review by UNC pathologists to ensure diagnostic accuracy. Systems for monitoring the frequency with which diagnoses are revised based on telepathology review are being developed, and these data are not currently available. However, anecdotally there has been a remarkable degree of consensus between Professor Liomba and UNC pathologists during telepathology sessions. Formal accreditation of the laboratory by DAIDS is now ongoing to support local participation in two phase III clinical trials for Kaposi sarcoma (KS), which are cosponsored by AMC and the AIDS Clinical Trials Group (ACTG). During the period reported, receipt of specimens was restricted to KCH and immediately adjacent clinics, but a timetable for receiving specimens from peripheral hospitals has been developed, as well as a fee schedule to allow revenue generation and ensure financial sustainability independent of external support.

**Table 2:** Immunohistochemistry stains being implemented in the Kamuzu Central Hospital pathology laboratory.

Stain	Use	Comment
**Ki-67**	Cellular proliferation marker used for several tumor types	Primarily used to distinguish Burkitt lymphoma from other non-Hodgkin lymphoma
**p16**	Marker for HPV in cervical, anal, and head & neck tumors	Currently used to assess HPV prevalence in head & neck squamous cell carcinomas
**LANA**	Marker for Kaposi sarcoma-associated herpesvirus	Confirmatory staining of Kaposi sarcoma allows patient enrollment into phase III clinical trials cosponsored by the AIDS Malignancy Consortium and AIDS Clinical Trials Group
**CD3**	Marker expressed on T-lymphocytes	Used to distinguish non-Hodgkin lymphoma subtypes
**CD20**	Marker expressed on B-lymphocytes	Used to distinguish non-Hodgkin lymphoma subtypes
**CD45**	Marker expressed on most hematopoietic cells	Used to distinguish hematopoietic from solid tumors when morphology is uncertain
**ER/PR**	Hormone receptor status assessment in breast cancer	Used to make tamoxifen and chemotherapy treatment decisions

HPV = human papillomavirus. LANA = latency-associated nuclear antigen. ER = estrogen receptor. PR = progesterone receptor.

### Data sources and analysis

At the time of specimen collection and submission, a standardized pathology requisition form is completed by requesting clinicians and provided to the laboratory, which includes basic details about the patient (age, gender, HIV status, antiretroviral therapy status, brief clinical details) and specimen (site, type, date of collection). Once received by the laboratory, each specimen is assigned a unique specimen number, and details from the requisition form, as well as specimen information and diagnostic conclusions after pathologist interpretation, are recorded in a secure institutional pathology database. Detailed clinical information about cancer stage, treatment, or outcomes is not available.

For this analysis, we included all specimens from the KCH pathology database received during the 20 months between July 1, 2011 and February 28, 2013. An attempt was made to identify all patients who were entered into the database more than once, by matching name, date of birth, or hospital number. Patients with more than one specimen were analyzed only once for patient-level analyses, and individual specimens were considered separately for specimen-level analyses. However, due to database limitations (e.g. missing data, name misspellings), it was not possible to ensure that all duplicated patients in the database were identified. Additionally, specimens were included only when a final diagnostic conclusion was recorded (including nondiagnostic conclusions).

Premalignant diagnoses were considered to be any atypical or dysplastic lesion without invasion (e.g. breast ductal carcinoma in situ, cervical intraepithelial neoplasia). Metaplastic lesions and benign lesions with low risk of malignant transformation, but without overt atypical or dysplastic features, were not considered premalignant. Malignant diagnoses were considered to be those with demonstrated histologic invasion or clearly malignant features by cytology. Other diagnosis categories were infectious (including bacterial, mycobacterial, fungal, and viral pathogens), other pathology not related to infection or cancer (e.g. goiter, branchial cleft cyst), normal or benign findings (e.g. lipoma, fibrocystic breast disease), and nondiagnostic.

Descriptive statistics were used to measure numbers of specimens assessed, patient characteristics, specimen types, and final diagnoses. Differences in proportions and means between patients with premalignant/malignant diagnoses and other diagnoses were assessed using chi-square and one-way analysis of variance (ANOVA) respectively. Bivariable and multivariable logistic regression were used to examine patient characteristics associated with premalignant or malignant diagnosis together, and also malignant diagnosis considered separately. All analyses were conducted using Stata version 12.1 (StataCorp, College Station, Texas USA). A two-sided alpha value of 0.05 was used to assess statistical significance. Records were excluded from analyses if these included variables for which data were missing.

## Results

**Figure 1:**
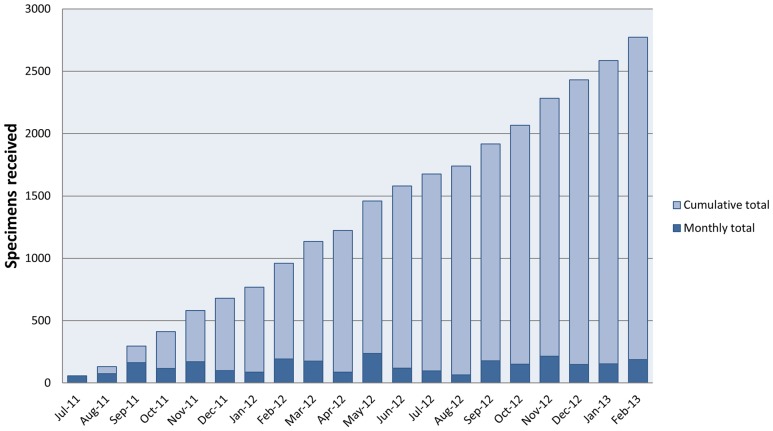
Specimens received in the Kamuzu Central Hospital pathology laboratory between July 1, 2011 and February 28, 2013.

The laboratory received 2772 specimens between July 1, 2011 and February 28, 2013 (Figure 1). Of these 2758 (99%) had a diagnostic conclusion entered and were included in these analyses. There were 119 duplicated patients identified, leaving 2639 unique individuals. Patient and specimen characteristics, as well as diagnosis categories, are shown in Table 3. Among unique patients, mean age was 38 years and 63% were female. HIV status was only routinely collected beginning in November 2012 and was therefore recorded for only 621 (24%) of patients. Of those with documented HIV status, 314 (51%) had unknown status, and 109 of 307 (36%) with known status were infected. Specimens from inpatient care settings comprised 43% of all cases. Histologic specimens comprised 91%, and cytologic specimens 9%. Specimens from gynecologic anatomic sites were most frequent. Malignancy accounted for 31% of all final diagnoses.

**Table 3:** Patient, specimen, and diagnosis characteristics in the Kamuzu Central Hospital pathology laboratory between July 1, 2011 and February 28, 2013.

PATIENTS	N = 2639
**Age (years)**	
Mean (SD)	38.4 (20.0)
Unknown	67 (2.5%)
**Gender**	
Female	1673 (63.4%)
Male	957 (36.3%)
Unknown	9 (0.0%)
HIV status*	
Infected	109/621 (17.6%)
Uninfected	198/621 (31.9%)
Unknown	314/621 (50.6%)

SPECIMENS	N = 2758
**Setting**	
Inpatient	1177 (42.7%)
Oupatient	1581 (57.3%)
**Type**	
Histology	2520 (91.4%)
Cytology	238 (8.6%)
**Site**	
Cervix/other gynecologic	847 (30.7%)
Head/neck (non-nodal)	340 (12.3%)
Lymph node	240 (8.7%)
Esophagus/stomach	178 (6.5%)
Breast	156 (5.7%)
Prostate	153 (5.5%)
Bowel/anal	145 (5.3%)
Bladder/kidney/urinary	89 (3.2%)
Eye	65 (2.4%)
Penis/testis/scrotum	37 (1.3%)
Other	508 (18.4%)

DIAGNOSES	N = 2758
Malignant	861 (31.2%)
Premalignant	178 (6.5%)
Infectious	407 (14.8%)
Other pathology	564 (20.4%)
Normal/benign	563 (20.4%)
Nondiagnostic	185 (6.7%)

SD = standard deviation. *HIV status routinely collected beginning November 2012.

Distribution of 171 confirmed premalignant diagnoses and 861 malignant diagnoses is shown in Table 4. Cervical dysplasia accounted for 88% of all premalignant diagnoses. Likewise, cervical cancer was the most common pathologically confirmed malignancy (n = 117, 14%), followed by lymphoma (n = 91, 11%), esophageal cancer (n = 86, 10%), sarcoma excluding Kaposi sarcoma (n = 75, 9%), and breast cancer (n = 61, 7%). HIV status was known for only 95 (11%) of malignant diagnoses overall. When HIV status was known, there were marked variations in HIV prevalence across cancer types, ranging from 9% for breast cancer to 81% for cervical cancer.

**Table 4:** Premalignant and malignant diagnoses in the Kamuzu Central Hospital pathology database between July 1, 2011 and February 28, 2013.

PREMALIGNANT	N = 178	HIV prevalence*
Cervix	156 (87.6%)	21/31 (67.7%)
Eye	13 (7.3%)	–
Other	9 (5.1%)	–
**MALIGNANT**	**N = 861**	
Cervix	117 (13.6%)	13/16 (81.2%)
Lymphoma	91 (10.6%)	4/13 (30.8%)
Esophagus	86 (10.0%)	2/8 (25.0%)
Sarcoma (non-Kaposi)	75 (8.7%)	2/9 (22.2%)
Breast	61 (7.1%)	1/11 (9.1%)
Head and neck	52 (6.0%)	–
Bladder	36 (4.2%)	–
Eye	36 (4.2%)	–
Prostate	36 (4.2%)	–
Kaposi sarcoma	29 (3.4%)	4/6 (66.7%)
Melanoma	26 (3.0%)	–
Ovary	19 (2.2%)	–
Penis	15 (1.7%)	–
Colorectal	13 (1.5%)	–
Other	169 (19.6%)	1/6 (16.7%)
**TOTAL**	**N = 1039**	

* Includes only specimens with known HIV status and reported only when at least five specimens with known HIV status were available.

Patient characteristics for pathologically confirmed premalignant or malignant diagnoses, versus other diagnoses, are shown in Table 5. Cases with premalignant or malignant diagnoses were significantly older than cases with other diagnoses (43 versus 36 years, p<0.0001). No significant differences were noted between these two groups with respect to gender (63% versus 65% female, p = 0.30) or HIV status (40% versus 35% infected, p = 0.29).

**Table 5:** Comparison of patient characteristics for premalignant/malignant diagnosis versus other diagnosis in the Kamuzu Central Hospital pathology laboratory between July 1, 2011 and February 28, 2013.

Variable	Premalignant/malignant	Other	p value
Age in years, mean (SD)*	43.0 (18.7)	35.6 (20.2)	<0.0001
Female*	649/1036 (62.6%)	1106/1712 (64.6%)	0.30
HIV-infected*	51/126 (40.5%)	70/202 (34.7%)	0.29

SD = standard deviation. *Includes only specimens with known patient age, gender, or HIV status.

Table 6 demonstrates bivariable and multivariable associations between patient characteristics (age, gender, HIV status) and final diagnosis of a premalignant or malignant condition, as well as final diagnosis of malignancy considered alone. Increasing age was consistently associated with a premalignant or malignant diagnosis [bivariable odds ratio (OR) 1.21 per decade increase (95% CI 1.16–1.26) among 2685 patients with known age; multivariable OR 1.24 per decade increase (95% CI 1.07–1.44) among 317 patients with known age, gender, and HIV status]. Female gender and HIV infection were not associated with malignancy or premalignacy, although estimation of HIV effects was limited by unknown status for most cases. Similarly, increasing age was associated with malignant diagnosis considered separately [bivariable OR 1.24 per decade increase (95% CI 1.19–1.29); multivariable OR 1.33 per decade increase (95% CI 1.14–1.56)], whereas HIV infection was not. However, female gender was associated with a reduced risk of malignancy considered separately [bivariable OR 0.61 (95% CI 0.52–0.72); multivariable OR 0.44 (95% CI 0.26–0.76)].

**Table 6:** Associations of patient characteristics with premalignant/malignant and malignant diagnosis in the Kamuzu Central Hospital pathology database between July 1, 2011 and February 28, 2013.

	Premalignant/malignant		Malignant only	
Variable	Bivariable OR (95% CI)*	Multivariable OR (95% CI)**	Bivariable OR (95% CI)*	Multivariable OR (95% CI)**
**Age, per decade**	1.21 (1.16–1.26)	1.24 (1.07–1.44)	1.24 (1.19–1.29)	1.33 (1.14–1.56)
**Female gender**	0.92 (0.78–1.08)	0.75 (0.45–1.27)	0.61 (0.52–0.72)	0.44 (0.26–0.76)
**HIV infection**	1.28 (0.81–2.03)	1.48 (0.92–2.40)	0.72 (0.43–1.20)	0.89 (0.52–1.52)

OR = odds ratio. CI = confidence interval. *Bivariable analyses include only specimens with known patient age (n = 2685), gender (n = 2748), or HIV status (n = 328). **Multivariable analyses include only 317 specimens with known patient age, gender, and HIV status.

To exclude bias introduced by a large number of nonmalignant specimens from cervical cancer screening programs, as well as bias introduced by unknown HIV status for the majority of cases, we conducted two additional sensitivity analyses. First, associations with malignant diagnosis were examined after exclusion of specimens from gynecologic sites. In these analyses, female gender was no longer associated with malignancy [bivariable OR 0.85 (95% CI 0.71–1.03); multivariable OR 0.85 (95% CI 0.45–1.61)], with estimates for age and HIV infection being otherwise similar. Second, HIV status was analyzed as a nondichtomous categorical variable (infected, uninfected, unknown), which resulted in bivariable and multivariable model estimates similar to results among patients with known HIV status only.

## Discussion

We describe the early experience after initiating a diagnostic pathology laboratory at a national teaching hospital in Lilongwe, a capital city and major urban center in sub-Saharan Africa where such services previously were lacking. It is hoped that these preliminary data will become more mature with fewer limitations over time. Nevertheless, we believe our experience is unique and informative, particularly given the broader momentum which is accumulating to increase pathology and cancer services throughout the region.

Numerous lessons have been learned during our early experience. First, a reliance on foreign pathologists is not sustainable, nor scalable. Virtual microscopy for us has proven valuable principally as an instrument for building collaborative relationships between pathologists and clinicians in Malawi and the United States, to foster educational sessions, exchange of ideas, and professional development activities. Researchers and trainees based in the United States have been stimulated by these sessions to develop projects for Malawi based on a clearer understanding of the burden of disease, and have opportunities to learn about the diverse histopathologic features of diseases rarely encountered in the United States, like KS and Burkitt lymphoma. Researchers and trainees based in Malawi have been stimulated to develop questions and pilot novel diagnostic assays and procedures appropriate for the setting, with support of colleagues in the United States. Telepathology has been an important tool for collaboration, rather than a primary mode by which diagnostic interpretations are rendered. Importantly, it cannot be a substitute for training a sufficient number of Malawian pathologists and laboratory technicians to provide essential diagnostic services, which is underscored by the fact that Professor Liomba himself has been the primary reader for more than 95% of specimens in a city of one million residents during recent months.

We are also learning important lessons about transitioning to laboratory independence. External start-up funds, together with the collaboration of long-term committed partners, can provide the essential support necessary to initiate diagnostic pathology services where these are lacking. It can also provide an environment in which there are opportunities for career development and meaningful collaborative relationships for Malawian pathologists and technicians, thus promoting retention of trained personnel which is essential for future independence and sustainability. With respect to financial sustainability, we are now undertaking a gradual expansion of services to receive specimens from peripheral hospitals beyond KCH and the immediately adjacent clinics, while remaining sensitive to laboratory workload and available staff. Development of locally appropriate fee schedules for revenue generation, as well as providing an environment in which Malawian investigators can develop research questions to pursue their own independent funding, are all examples of transitioning to a fully autonomous clinical and research laboratory in Lilongwe.

Apart from these essential lessons, our data provide some additional insights. First, increasing age was associated with an increased likelihood of malignancy. These results reinforce the urgency with which national cancer control programs should address the near doubling in cancer burden which is projected to occur over the next two decades, due to anticipated growth and aging of the population [1]. With respect to gender, the observed association between female sex and reduced risk of malignancy in our data is likely spurious, resulting from nearly one-third of all specimens coming from cervical cancer screening programs. This resulted in a differential contribution of nonmalignant specimens among women compared with men, for whom no cancer screening currently exists. When specimens from gynecologic sites were excluded, female gender was not associated with a reduced likelihood of malignancy, consistent with regional data which consistently demonstrate higher cancer incidence for women than men [2]–[4], [8].

We also found that HIV was not associated with malignancy. This is limited by the fact that HIV status was known for only slightly more than 10% of cases overall. However, HIV prevalence was 36% among cases with known status, which is more than three times the generalized HIV prevalence in Malawi of 10.6%, and similar to the high HIV prevalence in the KCH inpatient medical ward [9]–[11]. These discrepancies are indicative of persistently increased morbidity among HIV-infected individuals compared to the general population, and reflect challenges which remain despite scale-up of antiretroviral therapy (ART), such that 67% of Malawians needing ART now receive it [9]. The fact that two AIDS-defining cancers, cervical cancer and lymphoma, were the most common malignancies, partially reflects the impact HIV may be having on cancer incidence in Malawi.

There are significant limitations to this work. First, hospital-based data are subject to selection and referral bias, and do not represent cancer burden in Malawi in a manner analogous to population-based registration. KCH is one of two national teaching hospitals estimated to serve a referral population of 4–5 million. Patients have typically long symptomatic periods and advanced disease before being referred, a process which can take months or even years. As a result, patients at KCH represent a highly selected group, and many patients with cancer die before being referred. Second, in a resource-constrained environment, available services and practice patterns influence the case mix at a referral hospital pathology laboratory. For instance, KS is the most common cancer in Malawi,[3] but underrepresented in our results since it is treated typically on clinical grounds. Similarly, recent implementation of cervical cancer screening among high-risk women in HIV and sexually transmitted disease (STD) clinics directly influences the total number of cervical specimens, as well as HIV prevalence among confirmed cervical cancers. The capacity to biopsy many visceral sites also does not exist in Lilongwe, such that pathologically confirmed lung cancer will remain uncommon until clinical services needed to make this diagnosis improve.

Limitations of cancer registration in sub-Saharan Africa have been extensively discussed [12], [13]. Only 1% of African populations are estimated to be presently covered by cancer registries [13]. To our knowledge, there are only four countries in sub-Saharan Africa (Malawi, Uganda, Zimbabwe, and the Gambia), for which national population-based cancer incidence data are available [2]–[4], [13]–[15]. Only data from Uganda and Zimbabwe are included in the most recent IARC monograph detailing worldwide cancer incidence [16]. Taken together, available data demonstrate marked increases in cancer incidence over the last 10–20 years. However, data from these registries are limited by low rates of pathologic confirmation (18% in the Malawi registry), absent data on behavioral risk factors including tobacco and alcohol use, and absent HIV status. Along with ‘westernization’ of lifestyles, HIV is often cited as a reason for increasing cancer incidence. However, quantifying the effects of various risk factors on cancer burden remains difficult when these data are lacking in population-based registries, due to health system limitations throughout the region. For HIV, limited studies from sub-Saharan Africa have demonstrated an association between HIV and specific cancers, with many of these associations being similar to those observed in resource-rich settings [17]–[19].

Population-based registration also relies on retrospective standardized abstraction using multiple data sources. Despite having clear limitations, hospital-based data such as ours can supplement these efforts by providing information on pathologically confirmed cancers, including data on behavioral risk factors and HIV status provided by clinicians in real time. In the future, strategic and efficient design of laboratory requisition forms to capture key risk factor data without placing undue burden on clinicians, can allow these data to provide quantitative insights into the reasons underlying increasing cancer incidence as observed in population-based registries. Collection of cancer data during provision of clinical services also affords opportunities to link pathologically confirmed cancer diagnoses to longitudinal follow-up of patients to assess outcomes. Data with respect to cancer survival from population-based registries are exceedingly scarce from sub-Saharan Africa, and have been reported for only limited samples of cancer patients from Uganda, Zimbabwe, and the Gambia [20]–[23]. Additionally, hospital-based data from a major referral center like KCH can provide insights into local referral patterns and reasons for diagnosis and treatment delays. Such granular descriptions will be essential to inform cancer control efforts at the local level. Finally, the availability of tissue specimens in a national pathology laboratory linked to clinical data provides opportunities for correlative studies to elucidate tumor biology in this part of the world.

## Conclusions

A new pathology laboratory at a national teaching hospital in Malawi's capital has been well received and highly utilized by the entire medical community. The success of this endeavor over the first 20 months has depended on strong collaboration by multiple partners, funding from multiple sources with a view toward sustainability, and continuous adaptation and refinement of laboratory procedures to the available infrastructure and needs of the population. A robust platform for cancer care and research now exists in a setting where it did not previously, and can serve as a model for similar interventions throughout sub-Saharan Africa.
